# RNA-Seq of Human Neurons Derived from iPS Cells Reveals Candidate Long Non-Coding RNAs Involved in Neurogenesis and Neuropsychiatric Disorders

**DOI:** 10.1371/journal.pone.0023356

**Published:** 2011-09-07

**Authors:** Mingyan Lin, Erika Pedrosa, Abhishek Shah, Anastasia Hrabovsky, Shahina Maqbool, Deyou Zheng, Herbert M. Lachman

**Affiliations:** 1 Department of Genetics, Albert Einstein College of Medicine, Bronx, New York, United States of America; 2 Department of Psychiatry and Behavioral Sciences, Albert Einstein College of Medicine, Bronx, New York, United States of America; 3 Dominick Purpura Department of Neuroscience, Albert Einstein College of Medicine, Bronx, New York, United States of America; 4 Department of Neurology, Albert Einstein College of Medicine, Bronx, New York, United States of America; 5 Department of Medicine, Albert Einstein College of Medicine, Bronx, New York, United States of America; Rikagaku Kenkyūsho Brain Science Institute, Japan

## Abstract

Genome-wide expression analysis using next generation sequencing (RNA-Seq) provides an opportunity for in-depth molecular profiling of fundamental biological processes, such as cellular differentiation and malignant transformation. Differentiating human neurons derived from induced pluripotent stem cells (iPSCs) provide an ideal system for RNA-Seq since defective neurogenesis caused by abnormalities in transcription factors, DNA methylation, and chromatin modifiers lie at the heart of some neuropsychiatric disorders. As a preliminary step towards applying next generation sequencing using neurons derived from patient-specific iPSCs, we have carried out an RNA-Seq analysis on control human neurons. Dramatic changes in the expression of coding genes, long non-coding RNAs (lncRNAs), pseudogenes, and splice isoforms were seen during the transition from pluripotent stem cells to early differentiating neurons. A number of genes that undergo radical changes in expression during this transition include candidates for schizophrenia (SZ), bipolar disorder (BD) and autism spectrum disorders (ASD) that function as transcription factors and chromatin modifiers, such as *POU3F2* and *ZNF804A*, and genes coding for cell adhesion proteins implicated in these conditions including *NRXN1* and *NLGN1*. In addition, a number of novel lncRNAs were found to undergo dramatic changes in expression, one of which is *HOTAIRM1*, a regulator of several *HOXA* genes during myelopoiesis. The increase we observed in differentiating neurons suggests a role in neurogenesis as well. Finally, several lncRNAs that map near SNPs associated with SZ in genome wide association studies also increase during neuronal differentiation, suggesting that these novel transcripts may be abnormally regulated in a subgroup of patients.

## Introduction

Research on the biological basis of SZ and other neuropsychiatric disorders has been hampered by the inaccessibility of the human brain. However, the discovery of iPSC technology has the potential to address this problem by providing investigators with patient-specific neurons that can be used for disease modeling. In the past few years, investigators have taken advantage of this opportunity to establish iPSC lines in a variety of neuropsychiatric disorders including Rett Syndrome, Parkinson Disease, Amyotrophic Lateral Sclerosis, Familial Dysautonomia, and most recently, SZ [1]–[12]. In the study reported by Brennand *et al.* neurons derived from SZ-specific iPSCs showed diminished neuronal connectivity, reduced PSD95-protein levels and altered expression of WNT signaling pathways [1]. Similarly, we have also been developing iPSC lines from patients with SZ, a data set that includes patients with 22q11.2 deletions (in press).

In addition to their utility for disease modeling in neuropsychiatric problems, iPSCs can also be used to study early differentiating human neurons *in vitro* to gain insight into neurogenesis, which is particularly relevant to both SZ and ASD considering that both appear to have a neurodevelopmental basis [13]–[16].

With these aspects of disease pathogenesis in mind, we have analyzed the transcriptome of human neurons derived from iPSCs using RNA-Seq, a method that provides increased sensitivity with the capacity to detect low-copy transcripts, novel transcripts, lncRNAs, and splice isoforms [17]–[20]. The key role played by cell type-specific splicing in neuronal differentiation, particularly in genes coding for cell adhesion proteins, and the growing recognition that lncRNAs play a role neurogenesis lend further support for the value of deep sequencing transcriptome analysis [21]–[23].

Finally, a global, unbiased transcriptome analysis could help determine the biological significance of SNP markers associated with neuropsychiatric disorders identified in GWAS carried out in SZ, ASD, BD, many of which map to intergenic regions or deep within large introns where important regulatory lncRNAs may be found [24]–[26].

Our findings show that early differentiating neurons undergo an extraordinary array of quantitative changes in gene expression and in splice isoform generation, similar to that reported in differentiating human neurons derived from human embryonic stem cells (hESCs) [27]. In addition, we describe dramatic changes in expression of lncRNA genes during differentiation, one of which is *HOTAIRM1*, a cis-acting regulator of the *HOXA* cluster during myelopoiesis [28]. Contrary to previous reports suggesting that *HOTAIRM1* is not expressed in brain, a 54.6-fold increase in transcripts was detected in differentiating neurons, suggesting a novel role in neural differentiation. In addition, some GWAS SNPs found in SZ, BD and ASD mapped to lncRNA genes that are expressed in differentiating neurons, suggesting a role for these novel regulators in a subgroup of patients.

## Methods

This study was approved by the Internal Review Board of the Albert Einstein College of Medicine (protocol number 1996-013). Informed consent was obtained and the data were analyzed anonymously in accordance with the Declaration of Helsinki.

### iPSCs and neural differentiation

Details regarding the generation of iPSCs and the neuronal differentiation protocol used are described in Text S1.

### RNA-Seq

Total RNA was isolated from cells using the miRNeasy Kit (Qiagen) according to the manufacturer's protocol. An additional DNase1 digestion step was performed to ensure that the samples were not contaminated with genomic DNA. RNA purity was assessed using the ND-1000 Nanodrop. Each RNA sample had an A260∶A280 ratio above 1.8 and A260∶A230 ratio above 2.2. Briefly, total RNA (25 ng) was converted to cDNA using the NuGEN Ovation RNA-Seq System according to the manufacturer's protocol (NuGEN, San Carlos, CA, USA). The protocol employs a single primer isothermal amplification (SPIA) method to amplify RNA target into double stranded cDNA under standardized conditions that markedly deplete rRNA without preselecting mRNA. cDNA was then used for Illumina sequencing library preparation using Encore NGS Library System I. NuGEN-amplified double-stranded cDNA was fragmented into ∼300 base pair (bp) using a Covaris-S2 system. DNA fragments (200 ng) were then end-repaired to generate blunt ends with 5′ phosphatase and 3′ hydroxyls and adapters were ligated for paired end sequencing on Illumina HiSeq 2000. The purified cDNA library products were evaluated using the Agilent bioanalyzer and diluted to 10 nM for cluster generation in situ on the HiSeq paired-end flow cell using the CBot automated cluster generation system followed by massively-parallel sequencing (2×100 bp) on HiSeq 2000. We obtained 104-bp mate-paired reads from DNA fragments of average length of 250-bp (standard deviation for the distribution of inner distances between mate pairs is approximately 50 bp). iPSC and neuron RNA-Seq reads were separately aligned to the human genome (GRCh37/hg19) using the software TopHat (version 1.1.4) [29].

Splice junctions were automatically determined by TopHat, with the provided guidance of annotated gene models (GTF file) obtained mainly from Ensembl (http://www.ensembl.org). In our analysis, all three splice sites, “GT-AG”, “GC-AG” and “AT-AC” were considered. All splicing junctions supported by at least one high-quality mapped read were kept. The option for searching novel splice variants in Tophat was left on. The resulting alignment data from Tophat were then fed to an assembler Cufflinks (version 0.9.3) to assemble aligned RNA-Seq reads into transcripts [29]. Annotated transcripts were obtained from the UCSC genome browser (http://genome.ucsc.edu) and the Ensembl database; the category of transcripts was described at http://vega.sanger.ac.uk/info/about/gene_and_transcript_types.html. The number of transcripts in each category is listed in Table 1. Transcript abundances were measured in Fragments Per Kilobase of exon per Million fragments mapped (FPKM), which originated from the idea of RPKM (Reads per Kilobase per Million) [19]. To address the common issue in the assembly that a read may align to multiple isoforms of the same gene or multiple transcripts within the same genetic locus, maximum likelihood estimation was performed by Cufflinks based on a numerical optimization algorithm for calculating FPKM. Finally, the program Cuffdiff was used to define differential expression [29]. Instead of using transcript abundances computed separately by Cufflinks for each condition, Cuffdiff took alignment data from both iPSC and day 10 neurons, together with a list of human genome annotations (the same GTF file as used above, including both coding and non-coding transcripts) to infer expression differences at the level of transcript isoforms or primary transcripts or genes. Since we only had one replicate, the variance of FPKM was directly estimated from read counts using Poisson distribution, as described in detail at the Cufflinks website (http://cufflinks.cbcb.umd.edu/howitworks.html#hdif). In brief, the counts of reads that mapped to all nucleotides within a gene/transcript were assumed to follow Poisson distribution and then their mean was used as the variance. Student's t-test was then used to find significantly differentially expressed transcripts, with the test statistic derived from the log ratio of FPKM values in our two samples (see Cufflinks website for further details). To overcome the known bias in data normalization arising from a small number of highly expressed genes, we normalized our iPSC and neuronal data with total number of fragments mapped to the upper quartile of high expressed genes/transcripts rather than total mapped fragments [30].

**Table 1:** RNA-Seq Summary.

	Down-regulated from day 0 to 10	Up-regulated from day 0 to 10	Total number of genes
Total	5953	3055	50047
lincRNA	220	228	1443
miRNA	12	6	1809
Misc. RNA	18	30	1190
Proc. transcript	535	639	9062
Protein coding	4060	1808	21598
pseudogene	1052	319	10923
snoRNA	11	5	1523
snRNA	6	3	1951
rRNA	N/A	1	535
rRNA p. gene	22	2	179
scRNA p. gene	17	14	787

Abbreviations: lincRNA (long intergenic non-coding RNA); miRNA (microRNA); proc. (processed) (processed transcripts also known as lncRNA – long non-coding RNA); Misc. RNA (miscellaneous RNA, also includes mitochondrial RNA, mitochondrial transfer RNA, mitochondrial ribosomal RNA, polymorphic pseudogene); p. gene (pseudogene); snoRNA (small nucleolar RNA); snRNA (small nuclear RNA); rRNA (ribosomal RNA); scRNA (small cytoplasmic RNA).

Number (N) of RNA-Seq reads: Day 0: N of reads [pairs] 269,672,486 [134,836,243]; N of paired-match reads 121,620,928 [60,810,464]; N of single-match reads 39,276,866: Day 10: N of reads [pairs], 222,127,542 [111,063,771]; N of paired-match reads 115,280,074 [57,640,037], N of single-match reads 33,721,936.

### Validation by reverse transcriptase PCR (RT-PCR) and quantitative real time PCR (qPCR)

Selected genes and splice variants were validated by RT-PCR and qPCR using standard protocols that are described in detail in the Text S1. The primers used in these analyses are shown Table S1.

## Results

Complementary DNA was generated using RNA extracted from iPSCs and a 10 day cluster of neurons. Samples were amplified prior to paired-end deep sequencing as described in the methods section. More than 1×10^8^ pairs of reads were obtained for each sample, of which 74.3% and 82.3% had at least one end mapped to the reference genome (see legend Table 1 for details). The expression of 9008 genes was significantly altered as the iPSCs differentiated into neurons (q-value<0.05) (Table 1) (for a complete list of genes that showed significant changes in expression see Table S2). Of these, 5953 genes decreased in expression, while 3055 genes increased. Although the majority of these significantly altered genes were protein coding (4060 and 1808, respectively), approximately one-third were non-coding, findings similar to previous reports showing widespread expression in both coding and non-coding regions [31]–[34].

Consistent with the differentiation of iPSCs into neurons, substantial 10 to >1,000-fold decreases were detected in the expression of genes associated with pluripotency such as *OCT4 (POU5F1)*, *JARID2*, *NANOG*, and *LIN28A*, with a concomitant increase in expression of transcription factors associated with neural differentiation, including *POU3F2*, *MYT1L*, *NEUROD1*, and *MEF2C* (Table S3). *MYT1L*, *POU3F2*, along with *ASCL1*, are transcription factors capable of converting fibroblasts directly into neurons [35].

The most highly expressed genes in iPSCs were *TPT1*, *PTMA*, *NCL*, *NASP*, and *HSP90AB1*. *TPT1* (tumor protein, translationally-controlled 1) is involved in maintaining embryonic stem cell phenotype and interacts with *OCT4* and *NCL* (nucleolin), a nucleolar phosphoprotein involved in ribosomal maturation [36]–[38]. *PTMA* (prothymosin, alpha isoform 2) is over-expressed in a number of different cancers and interacts with histone acetyltransferases. *NASP* codes for nuclear autoantigenic sperm protein isoform 2 a histone H1 binding protein expressed in all dividing cells, inhibits proliferation and induces apoptosis in prostate cancer PC-3 cells, and is one of the hub genes involved in maintaining stem cell fate [39].

Among the most highly expressed genes in neurons were *TPT1, TUBA1B, HNRNPA2B1, C5orf13, RPS6, NCL, CANX, and RPL35A*. C5orf13 is neuronal protein 3.1 isoform A and is expressed in fetal and adult brains. Ribosomal protein S6 (*RPS6*) is phosphorylated in response to an array of growth factors and mitogens, a process that may be disrupted in Rett Syndrome [40]. However, among the top 25 genes that are the most highly expressed in neurons, all show higher levels of expression in iPSCs.

More interesting are the genes that show the most dramatic shifts in gene expression – both increases and decreases – during the transition from iPSCs to neurons. Among the transcripts that decreased radically were *SCGB3A2*, *POU5F1*, *DMKN*, *C14orf115*, *PRDM14*, *HLA-DPB2*, *POU5F1P1*, *TRIML2*, *MT1F*, *MT1X*, *CYP2S1*, and *IDO1*. *SCGB3A2* (secretoglobin, family 3A, member 2 precursor) maps to 5q32 and is a downstream target of the homeodomain transcription factor NKX2-1, which is critical for the development of lung, thyroid and ventral forebrain [41]. *DMKN* (dermokine isoform 1 precursor) is primarily expressed in skin and its expression in iPSCs is probably due to residual expression of fibroblast specific genes. *PRDM14* is a member of the PR domain and zinc finger proteins involved in transcriptional regulation. It is expressed in pluripotent stem cells and is involved in mouse embryonic stem cell (mESC) self-renewal, overlapping with *Nanog* and *Oct4* in its binding domain [42]. In addition, *PRDM14* regulates the expression of *OCT4* and is part of the network of genes involved in reprogramming human fibroblasts [43]. Finally, *POU5F1P1* may be a processed pseudogene or codes for a protein that binds to the nucleus that is believed to be involved in malignant transformation [44]–[46].

Considering the high level of expression in iPSCs and the rapid and dramatic decline that occurs during differentiation, some of these genes could be candidates for maintaining pluripotency or modulating cell type or region specific neural differentiation.

Another gene that showed one of the most significant fold-decreases during neuronal differentiation is *FZD5* (frizzled 5 precursor), a receptor for WNT5A, which is particularly interesting in the context of neuropsychiatric disorders because signaling through canonical WNT-mediated signal transduction appears to be involved in SZ and BD in subgroups of patients [1], [47]–[50]. Down regulation of FZD5 mediated signal transduction, similar to that described in differentiating hESCs, is consistent with a role in maintaining pluripotency [27].

*L1TD1* codes for LINE-1 type transposase domain containing 1. This gene appears to be a partner in the Oct4 interactome based on a protein affinity-based assay [51]. There is some evidence that L1 retrotransposition occurring selectively in neuronal progenitor cells could be a factor in brain development [52], [53].

Among the genes that showed the largest fold increase in expression during the transition from iPSCs to neurons was *CARTPT* (also known as *CART*), which codes for a neuropeptide involved in regulating food intake, reward and endocrine functions [54]. It is expressed in the hypothalamus and throughout the mesocorticolimbic dopamine reward system (nucleus accumbens shell, amygdala complex, extended amygdala and orbitofrontal cortex [55], [56].

Others include *STMN4* (stathmin-like 4), a member of the stathmin family that encode phosphoproteins involved in regulating the microtubule filament system in the brain, the AMPA2 receptor gene *GRIA2*, *ZIC4*, a zinc finger transcription factor expressed in the dorsal midline of the forebrain and in the boundary between the diencephalon and telencephalon, *SCN3A*, which codes for a sodium channel, voltage-gated, type III, alpha subunit and has been implicated in seizure disorders and neuropsychiatric problems, *EPHA3*, an ephrin receptor, the potassium channel encoding *KCNK2*, DCC (deleted in colorectal cancer) which codes for a receptor for the axon guidance protein netrin-1, and *SYT4* (synaptotagmin 4), a negative regulator of oxytocin release [57]–[59].

Several members from each of the four *HOX* families are among the most differentially expressed genes. *HOX* genes are involved in brain patterning in the development of forebrain, midbrain and hindbrain, and abnormal expression has been implicated in mental retardation, subtypes of ASD, and epilepsy [60]–[62].

Finally, previous work from our lab shows that the differentiation method we use results primarily in the development of glutamatergic neurons. This is reflected in the gene expression profile here, which shows that the glutamate transporter, *SLC17A6*, (*VGLUT2*) is among the most highly differentially expressed genes (Tables S2 and S3). By contrast, *SLC32A1* (VGAT, GABA vesicular transporter), *SLC6A20* (NET, norepinephrine transporter), *SLC6A4* (SERT, serotonin transporter), and *SLC18A1* and *SLC18A2* (VMAT1 and VMAT2A; vesicular monoamine transporters) were not expressed. The dopamine transporter gene, *SLC6A3*, (DAT1) was expressed, but at 1/50^th^ the level of *SLC17A6* (Table S2).

**Figure 1:**
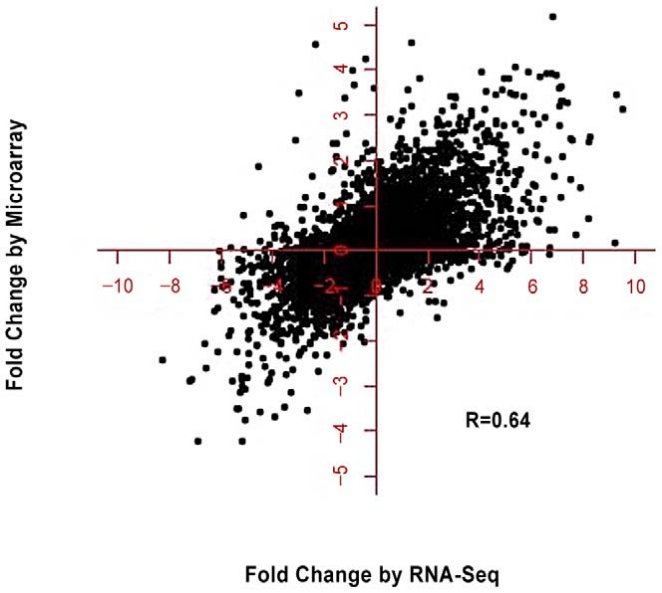
Comparison of fold-changes (log_2_-transformed) of gene expression between iPSCs and day 10 neurons as measured by RNA-Seq (x-axis) and Affymetrix microarray (y-axis).

The list of significantly altered genes was subjected to bioinformatics analysis using Gene Ontology (GO) to determine functional enrichment [63]. This showed changes in gene expression that were consistent with the conversion of pluripotent stem cells into terminally differentiated neurons (Table 2). In addition, there was high correlation (R = 0.64) between the results obtained with pre-amplified RNA using RNA-Seq and total RNA using standard microarray expression profiling (Fig. 1). The findings show that faithful amplification of RNA can be achieved, which is important for studying gene expression profiles in small numbers of neurons.

**Table 2:** Gene Ontology (GO) terms for differentially expressed genes.

GO TERMS FOR DOWN-REGULATED GENES		
	p-value	Benjamani
RNA processing	6.7E-56	3.1E-52
Translation	1.4E-44	3.2E-41
mRNA metabolic process	9.7E-36	1.5E-32
cell cycle	6.0E-35	6.9E-32
RNA Splicing	1.7E-33	1.6E-30
mRNA processing	7.2E-33	5.5E-30
RNP complex biogenesis	3.0E-31	2.0E-28
ribosomal biogenesis	3.8E-30	2.2E-27
mitotic cell cycle	4.6E-29	2.4E-26
cell cycle phase	2.6E-27	1.2E-24
DNA metabolic process	5.3E-27	2.2E-24
Cell cycle phase	1.2E-23	4.6E-21
translational elongation	1.7E-23	6.0E-21
RNA splicing via transester.	3.1E-23	1.0E-20
Nuclear mRNA splicing	3.1E-23	1.0E-20
rRNA metabolic process	7.3E-23	2.2E-20
ncRNA metabolic process	9.7E-23	2.8E-20

GO TERMS FOR UP-REGULATED GENES		
Neuron Differentiation	41.E-20	1.3E-16
Cell progection morph.	4.7E-18	8.2E-15
Pattern specification	1.3E-17	1.5E-14
Cell morphogenesis	2.1E-17	1.8E-14
Cell morph. Involved in diff.	5.8E-17	7.8E-14
Cell part morphogenesis	6.0E-17	6.5E-14
Cell adhesion	3.3E-16	1.7E-13
Biological adhesion	4.7E-16	1.9E-13
Regionalization	1.0E-15	3.9E-13
Cell morph. in neuron diff.	1.2E-15	4.3E-13
Axonogenesis	1.5E-15	4.9E-13
Ant/post pattern form.	2.2E-15	6.2E-13
Neuron projection morph.	3.1E-15	8.4E-13
Cellular component morph.	1.1E-14	2.7E-12
Neuron development	1.5E-14	3.5E-12

### lcnRNAs and lincRNAs

A substantial fraction of transcripts in mammalian genomes do not code for proteins. These include lncRNAs and lincRNAs (long intergenic non-coding RNAs), which are arbitrarily defined as greater than 200 bases in length, to distinguish them from functional small non-coding transcripts, such as microRNAs (miRNAs), piwi-interacting RNAs (piRNAs), and small nucleolar RNAs (snoRNAs).

LncRNAs (also known as processed transcripts) by definition are found within protein coding genes, overlapping with promoters, exons or introns in either sense or antisense orientations. LincRNAs, on the other hand, are found in intergenic regions. Two well-known lncRNAs are Xist, which initiates X-chromosome inactivation in females and *HOTAIR*, which is embedded within the *HOXC* locus, but has a trans-repressive effect on *HOXD* expression [64]–[66]. Other lncRNAs involved in regulating *HOX* gene expression are *HOTAIRM1* and *HOTTIP* [28], [67]. There is increasing evidence that lncRNAs are involved in stem cell pluripotency and brain development [22], [68]–[70]. Khalil et al showed that ∼20% of lincRNA bind to polycomb repressive complex 2 (PRC2), suggesting that many regulate gene expression at the chromatin level [71].

As shown in Table 1, 1622 non-coding genes (including lncRNAs and lincRNAs) significantly increased or decreased in expression during the transition from iPSCs to neurons. The most abundantly expressed lincRNAs in iPSCs are *MALAT1*, *SNHG6*, *SNORD87*, *CRNDE*, and *PVT1* (Table S4). *MALAT1* (metastasis-associated lung adenocarcinoma transcript 1) is also the most abundantly expressed lincRNA in differentiating neurons. It was initially identified in a screen for genes differentially expressed in metastatic non-small cell carcinoma [72]. MALAT1 appears to play a role in alternative splicing of pre-mRNAs since it localizes to nuclear speckles, dynamic structures that are essentially concentrations of splicing factors; it also recruits SR proteins, which participate in maturation of the splicosome [73]–[75]. *MALAT1* is up-regulated in the brains of PCP-treated mice and plays a role in hippocampal synaptic function [76]. Although the difference in expression between iPSCs and day 10 neurons was statistically significant, mRNA levels increased only ∼10%. However, to our surprise, there was a substantial difference in splice isoforms generated in iPSCs and neurons, including several that were specific to each (Fig. S1). The importance of this finding will require further study.

SNHG6/SNORD87 code for nucleolar RNAs, and *PVT1* is non-coding RNA oncogene that regulates and is regulated by *c-myc* [77]. *AL445670.1* is highly expressed in iPSCs and decreases 41-fold in neurons.

Among the lincRNAs that show the largest fold increases in neurons is *CRNDE*, a non-coding RNA of unknown function. *CRNDE* maps to 16q12.2, near the *IRX5* gene, a member of the Iroquois homeobox family, which are involved in brain patterning and neurogenesis [78], [79]. The close proximity to *IRX5* suggests that *CRNDE* may influence its expression.

Among the most highly expressed lncRNAs expressed in iPSCs are *XIST*, *AC00967.5/METTL5*, *SNHG5/SNORD50*, *STMN1*, and *RP11-509I21.1,RP11-509I21.2,TDGF1* (Table S5). Embryonic stem cells from female mice are characterized by the absence of *XIST* expression resulting in activation of both X-chromosomes, one of which is subsequently randomly inactivated upon differentiation. However in human ESCs and iPSCs, XIST expression is heterogeneous, resulting in various patterns of X-chromosome inactivation; lines that do not express *XIST* show expression of genes from both X-chromosomes [64], [80]. The iPSC line used in this RNA-Seq analysis was from a female.

AC00967.5 maps to 2q31.1 and overlaps with the *METTL5* (methyltransferase like 5) gene. It decreases 6-fold in neurons. *RP11-509I21.1* and *RP11-509I21.2* overlap with the OCT4 target *TDGF*1 (teratocarcinoma-derived growth factor 1; also known as *CRIPTO*) and is closely juxtaposed to the tumor suppressor gene *LRRC2* (leucine rich repeat containing 2) [81], [82]. A >1,000-fold decrease in expression of transcripts in this region was found during the transition from iPSCs to neurons. *TDGF1* is protein coding and is involved in NODAL signaling, which is a developmental program affecting midline, forebrain and left-right axis development [83]. Other lncRNAs that are highly expressed in iPSCs but decrease more than 50-fold in day 10 neurons include RP11-132A1.3, RP3-430A16.1, RP3-430A16.1, RP11-1144P22.1, RP11-509I21.1/RP11-509I21.2/TDGF1, RP11-498P14.2, RP11-366M4.3, AC005062.2/AC007001.3/MACC1. Some of these genes should be viewed as potential candidates for maintaining pluripotency.

Among the most interesting lncRNAs that show marked increases in expression in neurons is *HOTAIRM1*, which was suggested to be specific for myeloid cells [28]. However, we detected a 54.6-fold increase in transcripts in early differentiating neurons. Also, several other lncRNAs that map to *HOX* clusters showed dramatic increases in expression in day 10 neurons, including RP11-357H14.12 and AC036222.1, which map to the *HOXB* locus.

Other lncRNAs that show little or no expression in iPSCs, but significantly increase in early differentiating neurons, include *CTA-211A9.5/MIAT*, *RP11-732M18.3*, *AC018730*.*1*, *AC011306.2*, *RP11-65D13.1*, and *CTB-12O2.1*. *AC018730.1* is only ∼2kb upstream from *POU3F3* (*BRN-1*), a member of the brain-expressed POU family of transcription factors that is transcribed in the opposite orientation. Whether or not they share a common promoter, as we previously described for closely aligned genes transcribed in opposite orientations, remains to be seen [84]. *POU3F3* was not expressed in either the iPSC or neuron sample used in the RNA-Seq experiment.

### Validation by RT-PCR and q-PCR

**Figure 2:**
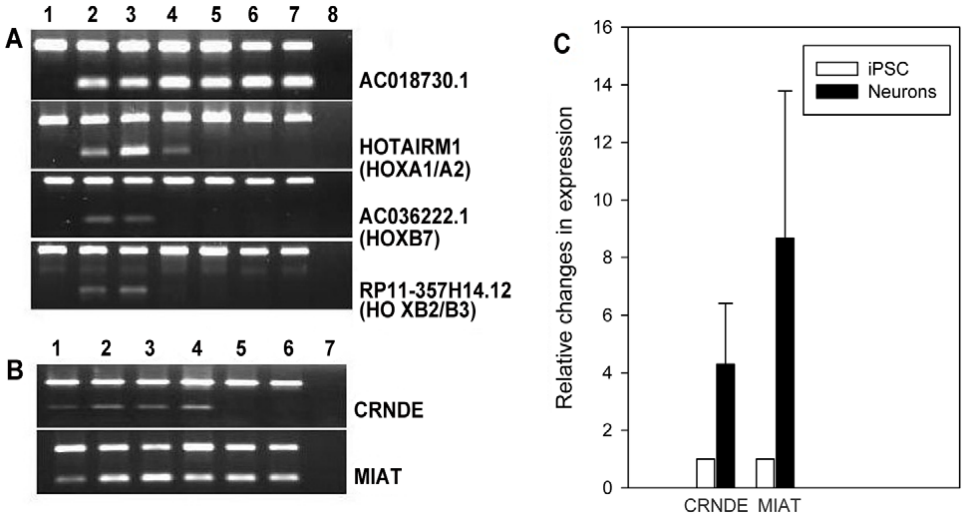
Validation by RT-PCR and qPCR in unamplified RNAs. **Panel A**, top band in each of the 4 sets is a beta-actin control; bottom band, specific transcripts. Lane 1, iPSCs; lane 2, day 14 neurons; lane 3, day 27 neurons; lanes 4 and 5, fetal brain (9 and 20 weeks, respectively); lanes 6 and 7, adult prefrontal cortex; lane 8, no RT (negative control). **Panel B**, same as A, but no 9 week fetal sample (lane 4 is 20 week fetal brain and lanes 5 and 6 are adult prefrontal cortex. **Panel C**, qPCR for *MIAT* and *CRNDE* in iPSCs (white bar) and day 14 neurons (black bar). Relative changes in gene expression were calculated using the 2^−ΔΔCt^ method with β2-microglobulin (β2M) as a reference gene. Mean increase in transcripts in neurons was statistically significant (p<0.05). Error bar is SD based on 3 or 4 replicates, each done in triplicate.

The expression of some of these non-coding elements was validated using RT-PCR and qPCR (Fig. 2). Considering the importance of *HOX* genes in neuronal migration and brain patterning, we focused primarily on those that map to the *HOX* loci since lncRNAs are known to be involved in their regulation. The RNA samples used in the validation were not amplified and were obtained from an independent set of neurons. *AC018730.1* transcripts, for example, could not be detected in iPSCs, but were abundant in both day 14 and 27 neurons. In addition, it is expressed in both fetal and adult brain samples. Similarly, *HOTAIRM1* RNA was detected in differentiating neurons but not iPSCs. However, in contrast to *AC018730.1*, expression in brain was restricted to the 9 week fetal sample. Expression of the other lncRNAs in the *HOX* gene loci - AC036222.1 and RP11-357H14.12 - was restricted to early differentiating neurons, and could not be detected in either fetal or adult brain. These findings suggest that the lncRNAs within the *HOXA* and *HOXB* loci are involved in early neurogenesis and brain development, and regulate expression of the *HOX* genes, and perhaps others, within specific developmental time frames.

We also validated the expression of *CRNDE* and *MIAT* by RT-PCR; lncRNAs that were highly expressed in both iPSCs and neurons. As seen in Fig. 2, there is a visible band in iPSCs that appears to increase in early differentiating neurons, similar to the RNA-Seq findings, which showed detectable transcripts in iPSCs that increased 3.7 and 3.6-fold in neurons (*CRNDE* and *MIAT*, respectively). Interestingly, *CRNDE* is expressed in fetal brain but transcripts could not be detected in adult brain samples, whereas *MIAT* is expressed in both.

qPCR was used to quantify the difference in expression in the un-amplified RNA samples. As seen in Fig. 2, a significant increase in expression was detected for both using qPCR that was in a range similar to that observed in the amplified RNA-Seq sample (*CRNDE*: 4.3-fold+/−2.1; *MIAT*: 8.7-fold+/−5.1; n = 3 or 4, each qPCR determination carried out in triplicate, p = 0.05 for both).

In addition to long non-coding RNAs, small non-coding RNAs were also found to be differentially expressed (Table 1). However, considering that the RNA amplification method we used does not accurately amplify small RNAs, the findings will not be discussed (a more definitive RNA-Seq analysis using a small RNA library prepared from an unamplified sample is currently being carried out).

### Pseudogenes

Some pseudogenes appear to be biologically functional with proposed effects on regulating gene expression through a variety of means, including acting as a miRNA decoy and producing endogenous short interfering RNAs [46], [84]–[89]. The expression of 1371 pseudogenes changed significantly during the transition from iPSCs to neurons (Table 1).

The most highly expressed in both iPSCs and day 10 neurons were RP11-442A13.1 and RP11-320L13.2, which map to 9q33.1 and Xq13.1, respectively (Table S6). The pseudogenes that showed the most significant fold changes upon differentiation were *TDGF3* (teratocarcinoma-derived growth factor 3, pseudogene; *CRIPTO-3*), which maps to Xq21.1, and RP11-492I2.1, which maps to an *ELAVL4* intron. *ELAVL4* transcripts increase significantly in our differentiating neurons and is induced in the brains of PCP treated rats [46], [76], [89], [90]. *TDGF3* is expressed in malignant cells and decreases upon differentiation of human teratocellular carcinoma cells. Among the more interesting pseudogenes that decrease in neurons are *POU5F1P4*, which is derived from *POU5F1*, and *TERF1P3* and *TERF1P4*. The parent gene (*TERF1*) codes for a component of the telomere nucleoprotein complex.

### Splice Variants

**Figure 3:**
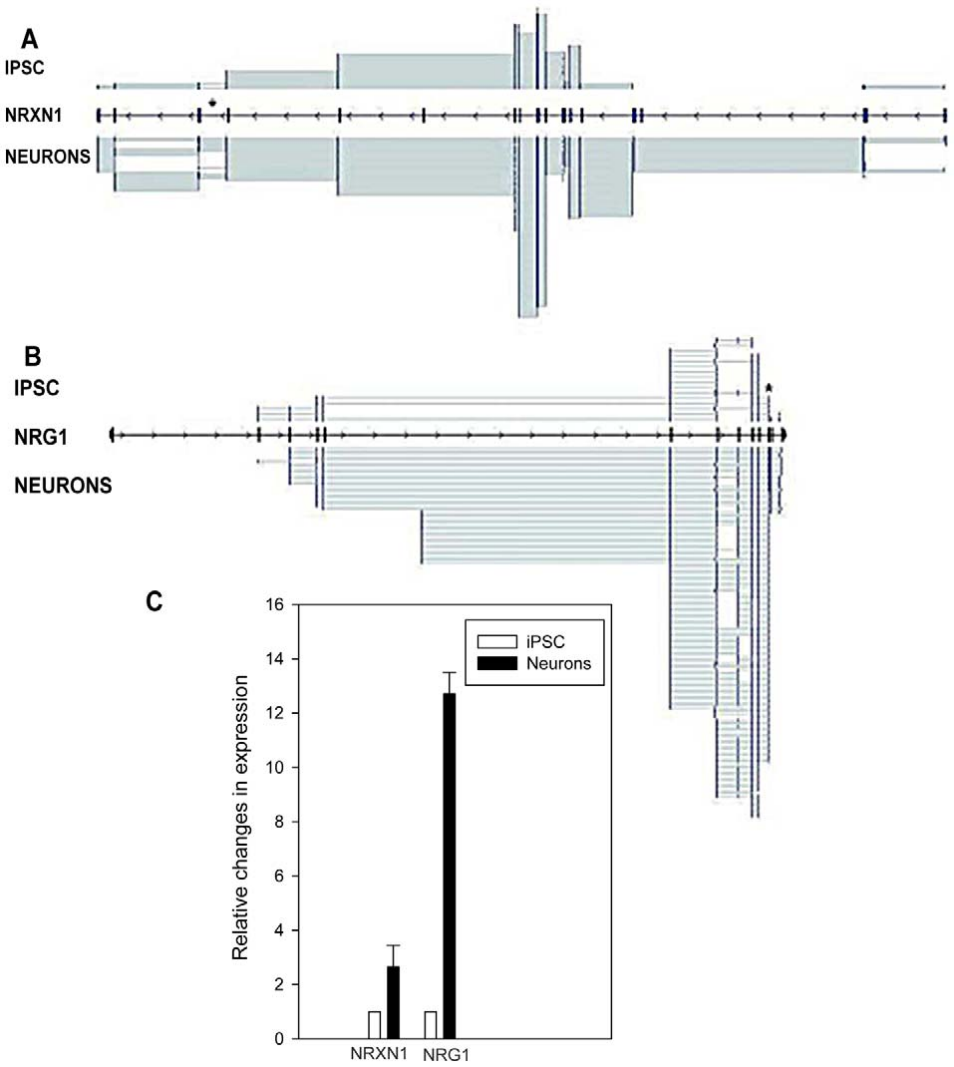
Splice variants in *NRXN1* (panel A) and *NRG1* (panel B). Asterisk in A shows splicing across exons 19, 20 and 21 (exons 20 and 21 are only ∼2 kb apart and appear superimposed in figure). Asterisk in B covers exons 10 and 11 in the NRG1 isoform HRG-β2b. **Panel C** shows qPCR and relative changes in gene expression calculated using the 2^−ΔΔCt^ method with β2-microglobulin (β2M) as a reference gene. Mean increase in transcripts in neurons was statistically significant (p<0.05). Error bar is SD based on 3 or 4 replicates, each done in triplicate.

Neuron specific splicing is regulated by several proteins, including NOVA1, a neuron specific RNA-binding protein, and the splicing inhibitor PTBP1 (polypyrimidine tract binding protein). *PTBP1* expression decreases ∼3-fold in day 10 neurons, while *NOVA1* expression increases ∼3-fold, consistent with their known effects on splicing in neuronal tissue. Our paired-end sequence reads allowed us to identify 143,586 splice junctions in iPSCs and 144,885 in day 10 neurons. Two examples are depicted in Fig. 3, which shows the changes in splice isoforms that occur in the SZ and ASD candidate gene, *NRXN1*. Expression across exons 19, 20 and 21 (asterisk in figure) is particularly interesting considering the importance of splice variants in this region, known as splice site 4, in determining glutamatergic vs GABAergic differentiation [91], [92]. We validated the apparent increase in expression across splice site 4 in this region by qPCR, which showed a 2.65-fold+/−0.79 increase in neurons (p<0.05).

We also show splice isoforms in the SZ candidate gene *NRG1*. Overall, *NRG1* transcripts increase ∼10-fold in day 10 neurons and a rich array of splice isoforms that change during differentiation is observed. We validated the splice variant formed between exons 10 and 11 in the NRG1 protein isoform HRG-β2b using qPCR (denoted by asterisk). As seen in the figure, a 12.7+/−0.79 fold increase was detected in differentiating neurons (p<0.05).

These findings show that RNA-Seq using amplified RNA will faithfully identify splice isoforms, and that dramatic changes in the overall pattern of alternatively spliced transcripts in candidate genes for neuropsychiatric disorders is an early event in neurogenesis.

### Relevance to neuropsychiatric disorders

As recently reported by Brennand et al., using neurons derived from patient-specific iPSCs is a valuable resource for gene expression profiling, which is the goal of our ongoing research as well [1]. However, important information related to SZ, ASD and BD pathogenesis can also be gleaned by studying molecular events occurring during human neurogenesis, even in the absence of patient vs control comparisons. For example, some of the most dramatic changes in gene expression in differentiating neurons in this study occur in genes involved in neuropsychiatric disorders that code for transcription factors and chromatin modifying enzymes. Subtle alterations in the expression of such genes during neurogenesis caused by genetic or environmental factors have the potential to induce long-lasting changes in neuronal and brain function. Among the SZ, BD, and ASD candidate transcription factors and chromatin modifiers that change dramatically in day 10 neurons are *POU3F2*, *MYT1L*, *RFX4*, *ZNF804A*, *SMARCA2*, and *NPAS3*, and the chromatin modifier *JARID2*, which regulates the polycomb repressive complex.

In addition, expression of a number of genes coding for proteins involved in cell-cell adhesion, synaptogenesis and trans-synaptic bridging that have been implicated in neuropsychiatric disorders increase in these early differentiating neurons. These include *NRXN1*, *NRXN3*, *NLGN1*, *CTNNA2*, *NCAM1* (and its modulator *ST8SIA2*), *CHL1*, *ELAVL4* and *PCDH9* [93]–[116].

Another way RNA-Seq data can be useful in understanding the underlying molecular and genetic basis of neuropsychiatric disorders is to identify non-coding elements transcribed in differentiating neurons that map to regions of the genome implicated by GWAS or CNVs. Many SNPs associated with neuropsychiatric disorders map to large introns or intergenic regions that are far removed from coding elements or known regulatory domains. This makes it difficult to appreciate the biological relevance of the association signal; presumably associated SNPs are in LD with disease-causing, biologically active variants.

One such SNP is rs893703, which maps to chromosome 3 (position139,250,649) within an *RBP1* (retinol binding protein) intron [117]. The SNP falls within a lncRNA that significantly increases in expression in differentiating neurons, *RP11-319G6.1* (Table S7). *RP11-319G6.1* is transcribed in the opposite orientation from *RBP1*, the latter of which decreases in differentiating neurons. *RP11-319G6.1* expression was validated in a different sample of differentiating neurons and is also expressed in fetal and adult brain (not shown). Whether or not *RP11-319G6* affects *RBP1* expression or other genes, and is a factor in the positive association found between rs893703 and SZ remains to be determined. Retinoids, it should be pointed out, play a role in brain development and have been implicated in SZ, although the evidence is not strong [118], [119].

Similarly the lncRNA *RP11-586K2.1*, which maps to chromosome 8 (89,044,236–89,749,363) contains a SNP (rs12527359) found to be associated with SZ and BD in a GWAS meta-analysis [120]. *RP11-586K2.1* increases ∼3.5-fold in differentiating neurons. It should be viewed as a potential candidate gene for this GWAS signal since the nearest coding genes to rs12527359 are *MMP16* (matrix metalloproteinase 16 isoform 1 and *RIPK2* (receptor-interacting serine-threonine kinase *2*), which are ∼400 kb–1,000 kb away, respectively.

A list of coding and non-coding genes that are differentially expressed in the RNA-Seq analysis that contain SNPs mapped in several SZ, BD and ASD GWAS studies are shown in Table S7 [120]–[123].

Finally, our transcriptome analysis could be useful in narrowing down candidate genes in well-defined large copy variants that have been found in SZ and ASD; coding genes, lncRNAs and lincRNAs that significantly change in differentiating neurons should be viewed as feasible candidates underlying the psychiatric manifestations of the copy variant. The CNVs we evaluated in this manner were 22q11.2 del, 15q11.2 del, 15q13.3 del, 16p11.2 dup, 17q12 del, 1q21.1 del, and 3q29 del [98], [124]–[132].

A total of 51 coding and non-coding genes in these CNVs are differentially expressed during early neurogenesis (Table S7). Of these a few appear to be particularly interesting, including the nicotinic cholinergic receptor subunit gene *CHRNA7*, and *QPRT*, which codes for quinolinate phosphoribosyltransferase, an enzyme involved in metabolizing an endogenous exitotoxin (quinolinate) implicated in Alzheimer Disease and Huntington Disease. Interesting two genes coding for proteins involved in phosphatidylinositol glycan (GPI) anchor biosynthesis – *PIGW* (chr17) and *PIGX* (chr3) – map to CNVs that are differentially expressed during neurogenesis. GPI anchors are used in cells to attach hydrophilic proteins to cell membranes. The hydrophobicity WNT3A, a signal transduction pathway believed to be involved in subsets of patients with SZ and BD, is increased by GPI-like anchors [133].

## Discussion

In this study we show that RNA from a limited sample can be reliably amplified preserving quantitative changes and splice isoform integrity. Several novel findings emerged, including the discovery that lncRNAs that map to the *HOXA* and *HOXB* gene loci are expressed early in neurogenesis, and that at least one, *HOXAIRM1*, is expressed in fetal brain. Considering the effect of *HOTAIRM1* on *HOXA* gene expression and myelopoiesis, our findings suggest a similar role in neurogenesis as well, but that remains to be determined experimentally. Similarly, the two other lncRNAs that map to *HOX* gene loci, RP11-357H14.12 and AC036222.1, that increase in differentiating neurons are excellent candidates for regulating *HOX* gene expression in the developing brain. The *HOX* genes play a role in brain development by determining rhombomere segmental identity, regional neural identity and anterior posterior patterning during early embryogenesis [60], [62]. Most of the work on the role of *HOX* genes on brain development has been carried out in drosophila and mouse. Using neurons derived from iPSCs will provide an opportunity to study the regulation of these genes, their targets, and their apparent lncRNA regulators in human tissue, *in vitro*.

Although analyzing the transcriptome during neurogenesis in humans is inherently valuable on its own, we are primarily interested in its application to neuropsychiatric disorders. These goals are not mutually exclusive since some of these disorders - notably SZ and ASD - have developmental origins and a primary defect in neurogenesis has been suggested. The RNA-Seq findings highlight the possibility of studying transcriptional/chromatin regulators involved in these disorders, and their downstream targets. Furthermore, unbiased genome wide transcriptome analysis has directed our attention to GWAS SNP signals that map to lncRNAs that increase in expression in differentiating neurons. Mutations causing aberrant expression of these non-coding RNAs represent very feasible candidates for the biologically significant variants that presumably lie in LD with disease-associated SNPs. Functional assays, such as studying the effects of knocking down expression of these lncRNAs in differentiating neurons, and targeted DNA resequencing in patients with SZ are warranted.

Establishing a role for non-coding RNAs in a subset of patients with neuropsychiatric disorders would add a new dimension to the genetic heterogeneity already known to underlie SZ, ASD and BD and would be consistent with the growing literature showing that this class of genes play a role in brain development and neuronal function. Two non-coding RNAs have previously been suggested to be involved in SZ in subgroups of patients; *DISC2* and *AK127244* [84], [134], [135]. *DISC2* is transcribed anti-sense to *DISC1* exon 9 and maps a few kilobases 3′ the breakpoint of the 1∶11 translocation that first targeted *DISC1* as a candidate gene in SZ. Antisense expression across exon 9 could conceivably modulate alternative splicing involving this exon. However, this has not been established experimentally. AK127244 is immediately upstream of *NRXN1*, and we previously showed that the two genes share a common promoter [84]. While copy variation affecting *NRXN1* is a fairly regular occurrence in patients with ASD and SZ, there have also been a few examples of copy loss in ASD in which only AK127244 is affected [136]. Although *AK127244* and *DISC2* transcripts were detected in the differentiating neurons we cultivated there was no significant change from that found in iPSCs.

Finally, RNA-Seq analysis revealed interesting aspects of alternative splicing that occur in differentiating neurons. Although this will be expanded in a subsequent paper, a few points are worth mentioning. For example, as shown in this paper, the generation of alternatively spliced isoforms in two candidate genes for SZ – *NRG1* and *NRXN1* – is a very early event in neurogenesis. This is consistent with the idea that abnormalities in neuronal migration and/or synaptogenesis, potential underlying factors in SZ, may begin as a gene expression programming problem in early differentiating neurons (either during fetal development and/or in the post-development brain).

In addition, novel splice isoforms were observed for the lncRNA *MALAT1*. *MALAT1* codes for an 8.7 kb transcript, which generates a 7.5 kb RNA that localizes to nuclear speckles, and a 61 nucleotide tRNA-like cytoplasmic molecule of unknown function produced by 3′ processing [137]. Splice isoforms have not been observed in studies showing Northern blots for *MALAT1*. However, spliced ESTs in the *MALAT1* gene locus that overlap with apparent spliced exons in our study have been isolated (UCSC Genome Browser, human EST track; Fig. S1). The different isoforms found in iPSCs and neurons suggest that *MALAT1* produces alternative transcripts in a cell type specific manner that could potentially modulate its role in pre-mRNA processing. Such an effect would be consistent with the role of nuclear speckles, cell type specific nuclear organization, and nuclear architecture on the differentiation of neurons and other cell types [74], [138]–[140].

## Supporting Information

Text S1 **Supplementary materials and methods. Detailed description of iPSC development and neural differentiation.**
(DOC)

Table S1 **PCR primers and antibodies used in experiments.**
(DOC)

Table S2 **File contains all differentially expressed genes significant at q<0.05. Columns show Gene Symbols, Position (Hg19), FPKM in iPSCs (Day_0_FPKM) FPKM in early differentiating neurons (Day_10_FPKM, fold change in expression (ln fold-change), Uncorrected p-values, and Type of primary gene.**
(XLS)

Table S3 **Top coding genes: absolute expression in iPSCs, day 10 neurons, and fold increase and decrease.**
(XLS)

Table S4 **Top lincRNAs.**
(XLS)

Table S5 **Top lncRNAs.**
(XLS)

Table S6 **Top pseudogenes.**
(XLS)

Table S7 **Differentially expressed genes that map to genome wide association (GWAS) SNPs and copy number variants (CNVs) found in schizophrenia, bipolar disorder and autism spectrum disorders.**
(XLS)

Figure S1 **Splice isoforms found in** ***MALAT1*** **transcripts.** Top panel, iPSCs; bottom panel, day 10 neurons. Continuous reads from adjacent exons depicted by thin line. Exon sequences are thick blue lines. Direction of transcription from left to right. Spliced ESTs on UCSC Genome Browser shown in black.
(TIF)
