# Supplementary material for: RNA-Seq of Human Neurons Derived from iPS Cells Reveals Candidate Long Non-Coding RNAs Involved in Neurogenesis and Neuropsychiatric Disorders
Source: PLoS One. 2011 Sep 7;6(9):e23356. doi: 10.1371/journal.pone.0023356 (PMC3168439; doi:10.1371/journal.pone.0023356)
Supplement: Text S1 — Supplementary materials and methods. Detailed description of iPSC development and neural differentiation. (DOC) [file pone.0023356.s001.doc]

**Materials and methods**

**Generating iPSCs**

The iPSC line used in this study was generated from fibroblasts obtained by skin biopsy from a healthy control female (29 years old). The study was approved by the Albert Einstein College of Medicine committee on clinical investigation. The

skin biopsy sample was transferred to a small Petri dish containing 2-3 ml Skin Fibroblast Media (SFM) consisting of RPMI 1640, 10% FBS, 1% pen/strep, 10ng/ml FGF2. The sample was incubated at room temperature for 15 minutes. Medium was carefully aspirated and replaced with 1-2 ml of collagenase type II solution (3mg/ml collagenase II dissolved in DMEM High Glucose [Worthington Biochemical Corp. Lakewood, NJ: GIBCO/Invitrogen, Carlsbad, CA]). The tissue was chopped into small pieces using 2 sterile scalpels, after which they were allowed to incubate at 37^o^C for 1­2 hours depending on size. The sample was then collected in a 15ml falcon tube and washed with SFM (serum free medium). Tissue was collected by centrifugation at 1200 rpm for 4 minutes. The pelleted sample was then suspended in SFM and plated in a T12.5 ml flask at 37^o^C in 5% CO_2_ for 3 days without changing medium or manipulation, to allow fibroblasts to adhere. Then cells were fed every 2 days with RPMI 1640 containing 10% FBS until a confluent culture was obtained (~3 weeks). The cells were reprogrammed into iPSCs using the Yamanaka transcription factors (*c-MYC, SOX2, Klf4,* and *OCT4 [POU5F1*]) driven by a retroviral LTR as previously described [1].

**Culture of Human iPSCs**

Stem cell lines were maintained on irradiated mouse embryonic fibroblasts (MEFs) in hES medium consisting of knockout (KO)-DMEM, 1mM pen/strep, 1 mM Gluta-MAX, 1X nonessential amino acids (NEAA), 55 μM β-mercaptoethanol (β-ME) (Gibco, Carlsbad, CA), 10% Knockout (KO) serum replacement (Invitrogen, Carlsbad, CA), 10% plasmanate (Talecris Biotherapeutics, Research Triangle Park, NC), and 10 ng/ml FGF2 (R&D Systems Inc., Minneapolis, MN). For maintenance of undifferentiated colonies, differentiated cells were manually removed and undifferentiated cells were passaged once a week. The iPSC line used in this experiment expressed the human embryonic stem cell markers SSEA-4, SSEA-3, TRA-1-60, and TRA-1-81, and had a normal karyotype – 46XX (J. Neurogenetics, in press; karyotyping carried out by Cell Line Genetics, Madison WI).

**Inducing iPSCs To Differentiate Into 3 Germ Layers**

iPSCs were washed once with DPBS, 2 ml per well. Then, 0.5 ml of CTK solution (CTK Solution (500μl 2.5% trypsin [25 mg in 1 ml, 1 mM HCl], 500 μl Collagenase IV [1 mg/ml working stock solution], 50 μl 0.1M CaCl_2_, 1 ml KO serum replacement, 3 mls H_2_O) was added and the cells were incubated for 5 min at 37^o^C. The wells were then washed once with 2 mls DPBS without CTK (note that CTK is harsh on cells and causes iPSC colonies to detach easily, care must be taken not to aspirate iPSC colonies during washes). Then 2 mls hES medium was added to the wells and remaining iPSC colonies were detached with a cell scraper, with care taken to avoid breaking up colonies. The colonies were then transferred to a 15 ml tube, and the colonies were allowed to settle to the bottom of tube for 5 min. The supernatant was carefully removed and fresh hES medium without FGF2 was added. The colonies were aliquoted with a 10 ml pipet to 6-well low-attachment cluster plates (2 mls per well). Plates were incubated for 2 days at 37^o^C, 5% CO_2_. Medium was changed as in the first section of methods. Thereafter, medium was changed every 2 days.

On day 8, chamber slides coated with 0.1% matrigel were prepared. The EBs were transferred to the chamber slides containing 0.5 ml hES and fed every other day by removing medium and adding fresh hES. After 8 additional days, immunocytochemistry for differentiated cell markers was carried out.

The line used in this RNA-Seq study differentiated into all 3 germ layers as shown by immunohistochemistry; alpha fetoprotein (endoderm), desmin (mesoderm), and MAP2 (ectoderm) and showed inactivation of exogenous *c-MYC, SOX2, Klf4,* and *OCT4* (J. Neurogenetics, in press).

Immunocytochemistry was carried as previously described [2,3]. A list of the antibodies used in the study is shown in Supplemental table 1.

**Neuronal Differentiation**

Neuronal differentiation for RNA-Seq experiment was induced in the following manner. iPSCs were treated with 1mg/ml dispase and detached using a cell scraper. Colonies were pooled together and washed in hES medium and grown as cellular aggregates (embryoid bodies; EBs) on Ultra Low Adherence 6-well plates (Costar catalog # 3471, Corning, Corning, NY) without FGF2 (day 0). EBs were maintained with a daily medium change for 4 days. Medium was changed by allowing the aggregates to settle by gravity, followed by removal of the supernatant and addition of fresh, pre-warmed medium. On day 4, aggregates were switched to a neural induction medium consisting of DMEM/F12, 1X N2 supplement (Invitrogen, Carlsbad, CA), 0.1mM NEAA, 2μg/ml heparin (Sigma-Aldrich Corp., St. Louis, MO), again omitting FGF2. Cultures were fed every other day by allowing aggregates to settle by gravity, removing supernatant, and replacing with fresh medium. On day 6, the EBs were transferred to laminin coated plates (20μg/ml [Roche]) to which they adhered. Feeding was carried out in neural induction medium every other day. Within 1-2 days, clusters of differentiated cells, including neurons, began to appear. Neurons were harvested on day 10 and day 32 by manual dissection.

To support glutamatergic differentiation, adherent colonies were treated on day 10 with WNT3A (100ng/ml) (R&D Systems, Minneapolis, MN) in neural induction medium. Cells were fed every other day for 2 weeks. On day 25, medium was changed to neuronal differentiation medium, which consists of Neurobasal medium, 1X N2 supplement, 2X B27, 0.1 mM NEAA, supplemented with cAMP (1 μM), BDNF, GDNF, and IGF1 (10 ng/ml each, PeproTech, Rocky Hill, NJ). The concentration of WNT3A was reduced to 10ng/ml.

**Alternative Neuronal Differentiation Protocol**

Neurons used for day 14 and day 27 validation experiments were derived from neural progenitor cells (NPCs) as described by Marchetto et al with slight modifications [4]. iPSCs were maintained in mTeSR1 medium (Stem Cell Technologies) for approximately 5-6 days. Colonies were checked for spontaneous differentiation under a dissecting microscope and were removed manually (on day 0). Before colonies displayed a dense center (5-6 days), the medium was changed to N2 media (DMEM/F12, 1X N2; Invitrogen), This was designated as day 0. The following day (Day 1), medium was changed to N2 plus 1μM Dorsomorphin (CALBIOCHEM). On day 2, EBs were created. Briefly, iPSCs were checked again for spontaneous differentiation and fresh N2 medium plus 1μM Dorsomorphin was added. Colonies were cut with a 5ml glass serological pipet using wide strokes to generate large fragments. A cell scraper was then used to detach remaining cells. EBs were aliquoted to a 6 well ultra-low attachment plate (Corning). On day 4, EBs were collected in a 15ml tube and allowed to settle via gravity for 5 minutes. Supernatant was removed and fresh N2 media plus 1μM Dorsomorphin was added. EBs were aliquoted to a new ultra-low attachment plate. From this point EBs were fed every 2 days.

On day 10, EBs were collected in a 15ml tube and allowed to settle by gravity for 5 minutes. Supernatant was removed and EBs were resuspended gently in NBF medium (DMEM/F12, 0.5X N2, 0.5X B27, 1% p/s) plus fresh 20ng/ml FGF2 (R&D Systems). Carefully with a 10ml pipet, EBs were aliquoted to a matrigel (BD Biosciences) plate. On day 12, plates were checked for rosette formation and fed with NBF medium plus fresh FGF2. Rosettes were fed every other day.

Rosettes were carefully excised on day 16 with a 26g needle and pooled in a 1.5ml tube. Accutase (ICT) was added to the rosettes for 3 minutes at 37^o^C. After incubation, rosettes were broken up with a 1ml pipet tip, centrifuged for 2 minutes at 1,000rpm, and washed once with 1X PBS (Invitrogen). The pellet was resuspended as single cells in NBF media with fresh FGF2 (20ng/ml) and aliquoted to Poly-L-Ornithine (Sigma)/Laminin (Roche) plates. NPCs were fed every other day.

Once NPCs reached 40-50% confluence, neural differentiation was induced by withdrawing FGF2. NBF media was supplemented with fresh growth factors as follows: WNT3a (100ng/ml) (R&D Systems), BDNF (10ng/ml), GDNF (10ng/ml), IGF-1 (10ng/ml) (PeproTech), and cAMP 1μM (Sigma). Cells were fed every other day.

**RT-PCR, qPCR, and Microarray Analysis**

#### Total RNA was extracted using a PicoPure® RNA Isolation Kit according to the manufacturer’s instructions (Arcturus Bioscience/Molecular devices, Sunnyvale, CA). An additional treatment with DNase1 (Qiagen, Valencia, CA) was included to remove genomic DNA. Reverse transcribed PCR (RT-PCR) was performed using a OneStep RT-PCR Kit (Qiagen, Valencia, CA) according to the manufacturer’s instructions. For quantitative real time PCR (qPCR) cDNA was synthesized using the iScript cDNA Synthesis Kit (Bio-Rad, Hercules, CA) according to manufacturer’s instructions. The cDNA was used as a template for quantitative PCR (qPCR), which was carried out using the ABI 7900HT Real-Time PCR System (Applied Biosystems, Foster City, CA). Each reaction consisted of cDNA, primers, and SYBR Green PCR Master Mix (Applied Biosystems, Foster City, CA) in an 8 μl volume (primers used in this study are shown in supplemental table 1). Melting curve analysis of target sequences showed that all primers used in this study generated amplicons consisting of a single peak, without primer-dimer artifacts. Primer concentrations were optimized prior to use in qPCR experiments. Standard curves were generated for each primer using fetal brain cDNA as a template to assess the quality of each qPCR experiment (slope of -3.3 indicates doubling at every cycle). Relative changes in gene expression were calculated using the 2^-∆∆Ct^ method with β2-microglobulin (β2M) as a reference gene. For gene expression profiling, an Affymetrix Human Gene 1.0 ST Array was used. RNA samples used in the array passed quality control assessed by Agilent 2100 Bioanalyzer. Hybridization was carried out according to the manufacturer’s instructions on duplicate samples and analyzed using the LIMMA algorithm [3]. At a p-value of 0.05, we detected 645 and 383 genes up- and down-regulated between day 0 and day 10, respectively, and 146 and 280 genes up- and down-regulated between day 10 and day 32. In total, 1207 genes were found to be differentially expressed using LIMMA, and these genes were hierarchically clustered to form 5 groups. Microarray data have been deposited in the Gene Expression Omnibus (GEO; accession number GSE26629) and additional details can be found in Pedrosa et al (in press, Journal of Neurogenetics).

References

1. Takahashi K, Tanabe K, Ohnuki M, Narita M, Ichisaka T, et al. (2007) Induction of pluripotent stem cells from adult human fibroblasts by defined factors. Cell 131(5): 861-872. 10.1016/j.cell.2007.11.019.

2. Davidkova G, Carroll RC. (2007) Characterization of the role of microtubule-associated protein 1B in metabotropic glutamate receptor-mediated endocytosis of AMPA receptors in hippocampus. J Neurosci 27(48): 13273-13278. 10.1523/JNEUROSCI.3334-07.2007.

3. Huangfu D, Osafune K, Maehr R, Guo W, Eijkelenboom A, et al. (2008) Induction of pluripotent stem cells from primary human fibroblasts with only Oct4 and Sox2. Nat Biotechnol 26(11): 1269-1275. 10.1038/nbt.1502.

4. Marchetto MC, Carromeu C, Acab A, Yu D, Yeo GW, et al. (2010) A model for neural development and treatment of rett syndrome using human induced pluripotent stem cells. Cell 143(4): 527-539. 10.1016/j.cell.2010.10.016.

-
