# Supplementary material for: RNA-Seq of Human Neurons Derived from iPS Cells Reveals Candidate Long Non-Coding RNAs Involved in Neurogenesis and Neuropsychiatric Disorders
Source: PLoS One. 2011 Sep 7;6(9):e23356. doi: 10.1371/journal.pone.0023356 (PMC3168439; doi:10.1371/journal.pone.0023356)
Supplement: Table S1 — PCR primers and antibodies used in experiments. (DOC) [file pone.0023356.s002.doc]

**SUPPLEMENTARY TABLE 1: PCR PRIMERS**

Gene primers size (bp) anneal temp

Β-ACTIN TCACCACCACGGCCGAGCG 351 53

TCTCCTTCTGCATCCTGTCG

B2M GCTCGCGCTACTCTCTCTTT 140 57

CAATGTCGGATGGATGAAAC

NANOG TACCTCAGCCTCCAGCAGAT 215 53

CATCCCTGGTGGTAGGAAGA

OCT4 AGATATGCAAAGCAGAAACC 236 47

ATCCTCTCGTTGTGCATAGT

EndoOCT4F GACAGGGGGAGGGGAGGAGCTAGG 144 49

EndoOCT4R CTTCCCTCCAACCAGTTGCCCCAAAC

POU3F2 GACCTTTGCAGGCGAGTAAC 196 49

TCAGGAAGCTGCATTTTGTG

VGLUT2 ATCTTTTAGGTGCAATGGAA 191 45

CACAGCAGATAGCATACCAA

WNT3A AGTGACACGCTCATGTGCAGAA 129 52

AGACACCATCCCACCAAACTCGAT

ZNF804A CCAGCTCTCACCAGAACCTC 215 49

GGTTGCAAAGGGATGACAGT

MIAT-F TGTCTCCATTTGCTCAGTGC 189 53

MIAT-R TCAGGATGGTGCACTCTCAG

132A-F TTCACACTTGCAGCAGATCC 245 53

132A-R TCATGTCTCCTGGGTCCTTC

RP11-132A1.3

187F CAAAGGACAACGGAGGAAGA 178 53

187R GCGACAAGTCAGACCTAGCC

AC018730.1

14.7F TAGCGCCTCATCGCTCTTAT 220 53

14.7R TCAGTTCCACTCGGTTGTCA

RP11-357H-14.7 HOXB5, HOXB6

14.12F TGGGAAATGGACAGGAACTC 181 53

14.12R AGAAGCATGGAAGCCTGAAA

RP11-357H14.12

AC0-F TGCGAGAGAATCTTGTTCAGC 100 53

AC0-R GGAAGCAGCTCTGTGGTCTC

AC036222.1 HOXb7 locus

NRXN1SS4F CATCGCCATTGAAGAATCCA 137; 227 49

NRXN1SS4R GAAGATTGTGAGCTGACGCC

NRG1splicevalF GAGACATCCTTTTCCACCAG 140 53

NRG1splicevalR GACATCACGATTACAGAGTG

CRNDE-F GATGACTCATTG TGAGTGCTAG 125 53

CRNDE-R CCATGTTCTTTGCATCTAGATT

HAIRF GCCAGAAACCAGCCATAGTC 193 53

HAIRR GAAAACGCAGCATGTAAGCA

HOTAIRM1

319F GAATTCTCTGCAGGCCTTTG 172 53

319R TGTTCACAAACACACCCACA

RP11-319G6.1

117F GCAGGGCAAAGAGAAAAGTG 229 53

117R ACAGCCGGTGAAGATTTGAG

AC011754.1

586F GGCTGGATGAAATGAAAGGA 209 53

586R GGTTTGTGGCTTGCATTTTT

RP11-586K2.1

926F TTTGAAAAGCCCTTCCTCTG` 171 53

926R ATCGTCATCTGGGAGAGCAG

AC092676.3

CNAP2F GGATGCTCTACAGCGACACA 166 53

CNAP2R TCTCCATTCCAATCCAGAGG

CNTNAP2

**VIRAL PRIMERS FOR IPSC REPROGRAMMING FACTORS***

pMXslL3205 CCCTTTTTCTGGAGACTAAATAAA

exoOCT4F CCCCAGGGCCCCATTTTGGTACC

exoSOX2-S691 GGCACCCCTGGCATGGCTCTTGGCTC

exoKLF4-S1128 ACGATCGTGGCCCCGGAAAAGGACC

exoMYC-S1011 CAACAACCGAAAATGCACCAGCCCCAG

*(iPSC reprogramming factors PCR products generated using virus specific primer – pMXs – along with factor-specific primers)

**ANTIBODIES**

1^o^ Antibody 2^o^ Antibody

Anti-OCT-4 [POU5F1] Millipore cat#MAB4419 DyLight 488-conjugated AffiniPure Goat Anti-Mouse IgG (H+L) Jackson Immuno Research cat#115-485-003

Phycoerythrin (PE) anti-human TRA-1-60 N/A

eBioscience cat#12-8863

Phycoerythrin (PE) anti-human TRA-1-81 N/A

eBioscience cat#12-8883

Alexa Fluor 488 anti-mouse/human SSEA-3 Alexa Fluor 488 goat anti-rat IgM (μ chain)

eBioscience cat#53-8833 Invitrogen cat#A21212

FITC Mouse anti-SSEA-4 BD Biosciences Alexa Fluor 488 rabbit anti-mouse IgG

Cat# 560126 (H+L) Invitrogen cat#A11059

Anti-human/mouse α-Fetoprotein Alexa Fluor 488 rabbit anti-mouse IgG

R&D Systems cat#MAB1368 (H+L) Invitrogen cat#A11059

Anti-Desmin (Muscle Cell Marker) Ab-1 Alexa Fluor 488 rabbit anti-mouse (clone D33) IgG Thermo Scientific cat#MS-376-S1 (H+L) Invitrogen cat#A11059

anti-Synapsin (rabbit polyclonal, 1:1000

Chemicon/MIllipore, Temecula, CA) Donkey anti-rabbit Jackson Immunoresearch (JI), West Grove, PA).

anti-VGLUT2 (mouse monoclonal, 1:1000 Donkey anti-mouse, JI

Synaptic Systems Goettingen, Germany),

cat#135403

anti-MAP2 (mouse monoclonal, 1:1000, Donkey anti-mouse, JI

Chemicon.

anti GABA (mouse or rabbit, 1:5000 Donkey anti-mouse, anti rabbit, JI

Sigma Aldrich, St. Louis, MO).

Sheep/Tyrosine Hydroxylase Pel-Freez

Cat#P60101

anti-Synapsin (rabbit polyclonal, 1:1000 Donkey anti rabbit, JI

Chemicon/MIllipore, Temecula, CA)

All secondary Ab labeld with Cy3, FITC, or Cy5, Jackson Immunoresearch, West Grove, PA.

DAPI nuclear stain: ProLong Gold antifade reagent with DAPI, Invitrogen cat#P-36931
