# Supplementary figures and images for: RNA-Seq of Human Neurons Derived from iPS Cells Reveals Candidate Long Non-Coding RNAs Involved in Neurogenesis and Neuropsychiatric Disorders
Source: PLoS One. 2011 Sep 7;6(9):e23356. doi: 10.1371/journal.pone.0023356 (PMC3168439; doi:10.1371/journal.pone.0023356)

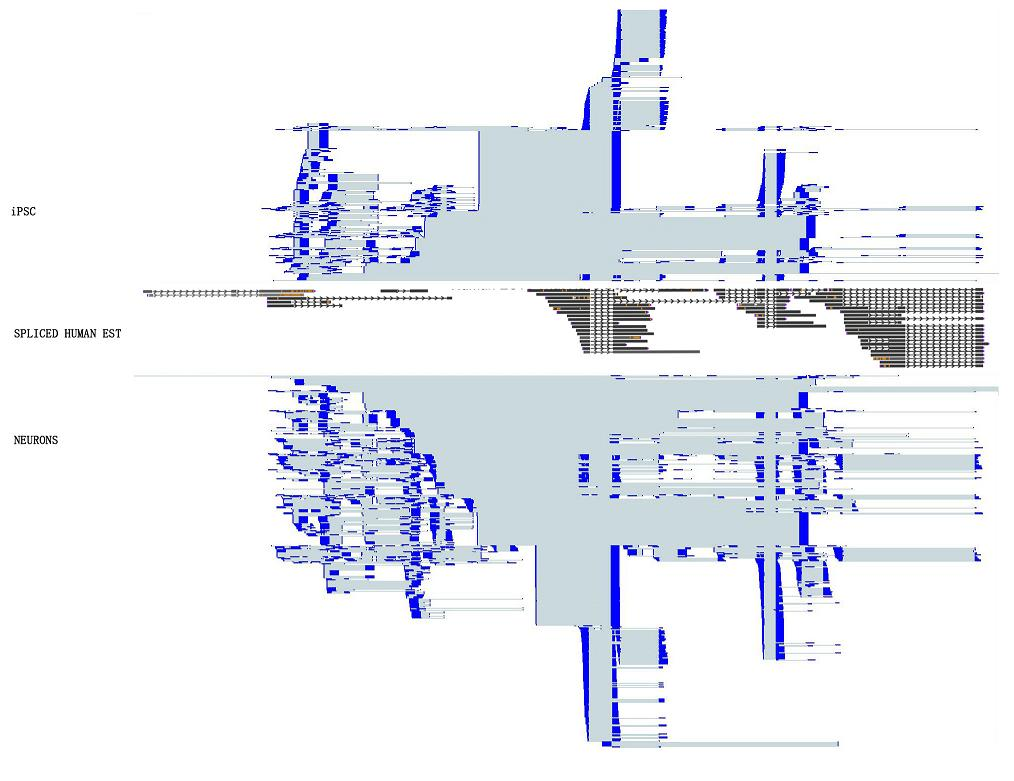

Supplement: Figure S1 — Splice isoforms found in MALAT1 transcripts. Top panel, iPSCs; bottom panel, day 10 neurons. Continuous reads from adjacent exons depicted by thin line. Exon sequences are thick blue lines. Direction of transcription from left to right. Spliced ESTs on UCSC Genome Browser shown in black. (TIF) [file pone.0023356.s009.tif]
